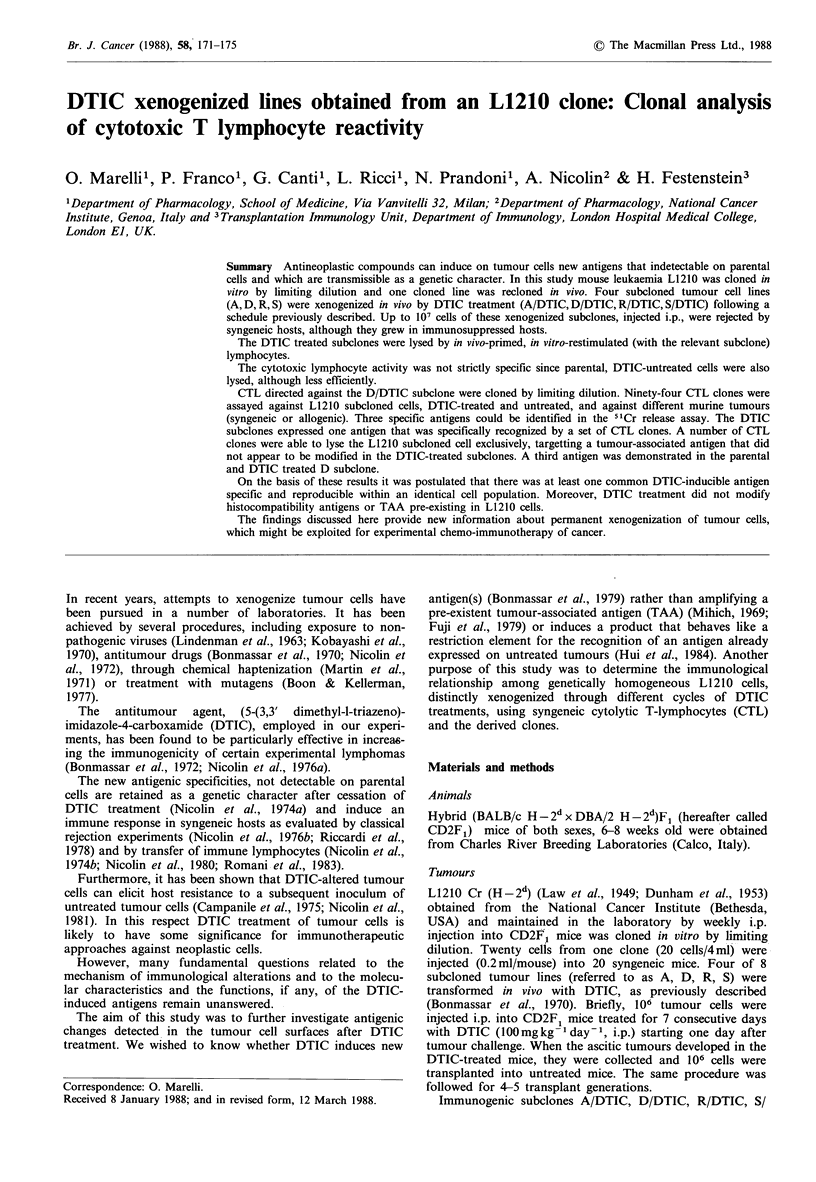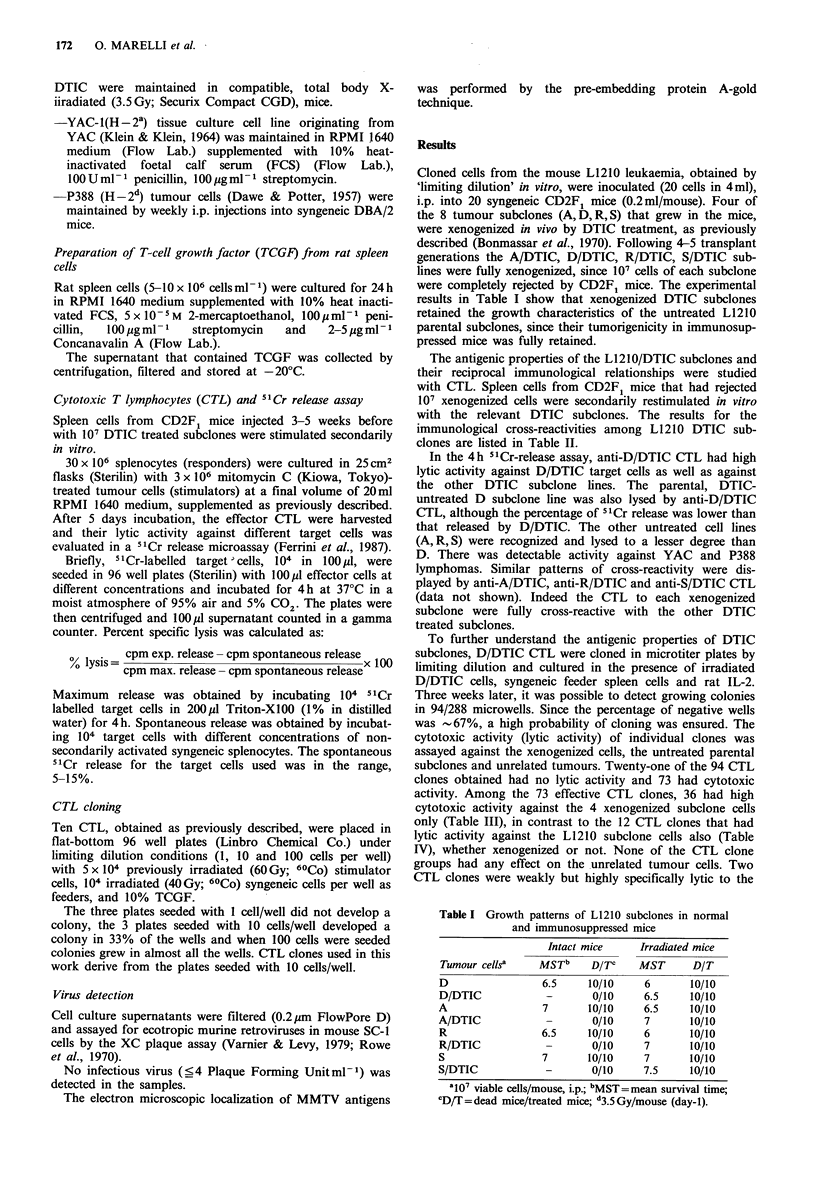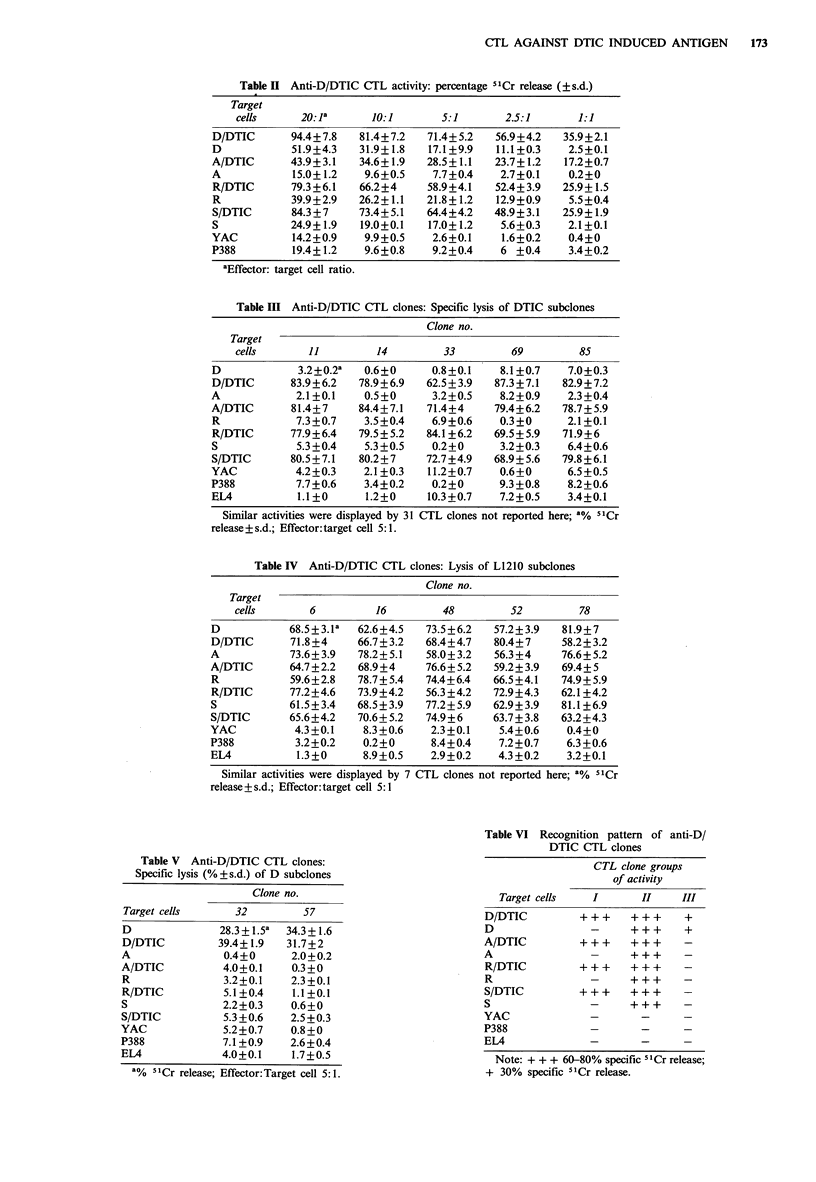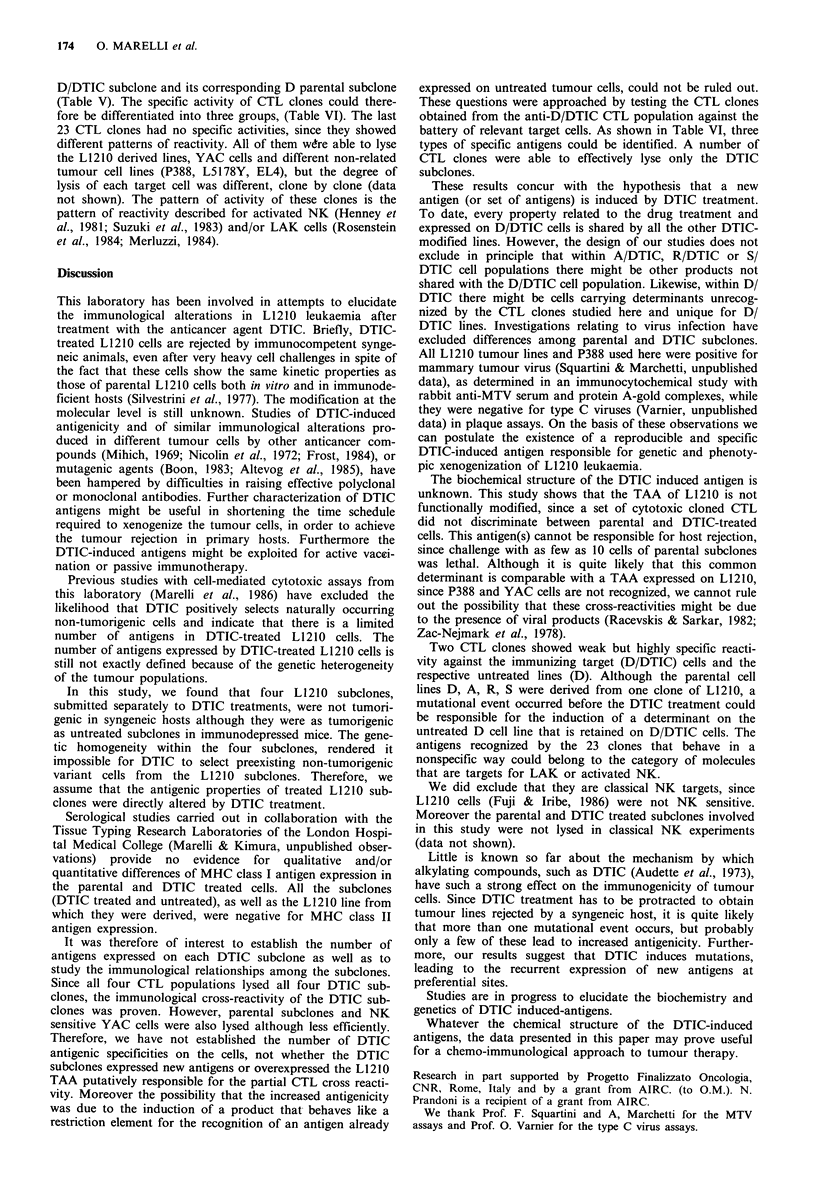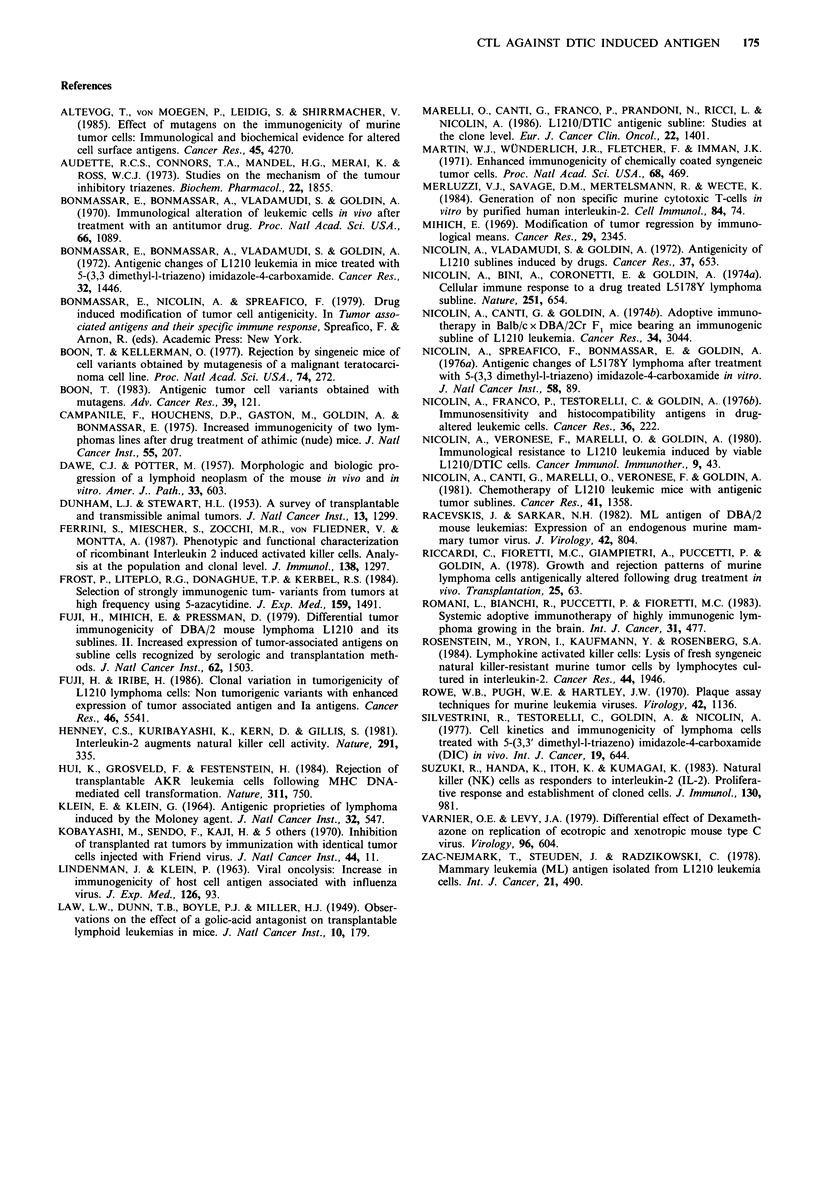# DTIC xenogenized lines obtained from an L1210 clone: clonal analysis of cytotoxic T lymphocyte reactivity.

**DOI:** 10.1038/bjc.1988.186

**Published:** 1988-08

**Authors:** O. Marelli, P. Franco, G. Canti, L. Ricci, N. Prandoni, A. Nicolin, H. Festenstein

**Affiliations:** Department of Pharmacology, School of Medicine, Milan.

## Abstract

Antineoplastic compounds can induce on tumour cells new antigens that undetectable on parental cells and which are transmissible as a genetic character. In this study mouse leukaemia L1210 was cloned in vitro by limiting dilution and one cloned line was recloned in vivo. Four subcloned tumour cell lines (A,D,R,S) were xenogenized in vivo by DTIC treatment (A/DTIC, D/DTIC, R/DTIC, S/DTIC) following a schedule previously described. Up to 10(7) cells of these xenogenized subclones, injected i.p., were rejected by syngeneic hosts, although they grew in immunosuppressed hosts. The DTIC treated subclones were lysed by in vivo-primed, in vitro-restimulated (with the relevant subclone) lymphocytes. The cytotoxic lymphocyte activity was not strictly specific since parental, DTIC-untreated cells were also lysed, although less efficiently. CTL directed against the D/DTIC subclone were cloned by limiting dilution. Ninety-four CTL clones were assayed against L1210 subcloned cells, DTIC-treated and untreated, and against different murine tumours (syngeneic or allogenic). Three specific antigens could be identified in the 51Cr release assay. The DTIC subclones expressed one antigen that was specifically recognized by a set of CTL clones. A number of CTL clones were able to lyse the L1210 subcloned cell exclusively, targetting a tumour-associated antigen that did not appear to be modified in the DTIC-treated subclones. A third antigen was demonstrated in the parental and DTIC treated D subclone. On the basis of these results it was postulated that there was at least one common DTIC-inducible antigen specific and reproducible within an identical cell population. Moreover, DTIC treatment did not modify histocompatibility antigens or TAA pre-existing in L1210 cells. The findings discussed here provide new information about permanent xenogenization of tumour cells, which might be exploited for experimental chemo-immunotherapy of cancer.


Br. J. Cancer (1988), 58, 171–175 © The Macmillan Press Ltd., 1988

### DTIC xenogenized lines obtained from an L1210 clone: Clonal analysis of cytotoxic T lymphocyte reactivity

O. Marelli[1], P. Franco[1], G. Canti[1], L. Ricci[1], N. Prandoni[1], A. Nicolin[2] & H. Festenstein[3]

[1]*Department of Pharmacology, School of Medicine, Via Vanvitelli 32, Milan;* [2]*Department of Pharmacology, National Cancer Institute, Genoa, Italy and* [3]*Transplantation Immunology Unit, Department of Immunology, London Hospital Medical College, London E1, UK.*

**Summary** Antineoplastic compounds can induce on tumour cells new antigens that indetectable on parental cells and which are transmissible as a genetic character. In this study mouse leukaemia L1210 was cloned *in vitro* by limiting dilution and one cloned line was recloned *in vivo*. Four subcloned tumour cell lines (A, D, R, S) were xenogenized *in vivo* by DTIC treatment (A/DTIC, D/DTIC, R/DTIC, S/DTIC) following a schedule previously described. Up to $10^7$ cells of these xenogenized subclones, injected i.p., were rejected by syngeneic hosts, although they grew in immunosuppressed hosts.

The DTIC treated subclones were lysed by *in vivo*-primed, *in vitro*-restimulated (with the relevant subclone) lymphocytes.

The cytotoxic lymphocyte activity was not strictly specific since parental, DTIC-untreated cells were also lysed, although less efficiently.

CTL directed against the D/DTIC subclone were cloned by limiting dilution. Ninety-four CTL clones were assayed against L1210 subcloned cells, DTIC-treated and untreated, and against different murine tumours (syngeneic or allogenic). Three specific antigens could be identified in the $^{51}Cr$ release assay. The DTIC subclones expressed one antigen that was specifically recognized by a set of CTL clones. A number of CTL clones were able to lyse the L1210 subcloned cell exclusively, targetting a tumour-associated antigen that did not appear to be modified in the DTIC-treated subclones. A third antigen was demonstrated in the parental and DTIC treated D subclone.

On the basis of these results it was postulated that there was at least one common DTIC-inducible antigen specific and reproducible within an identical cell population. Moreover, DTIC treatment did not modify histocompatibility antigens or TAA pre-existing in L1210 cells.

The findings discussed here provide new information about permanent xenogenization of tumour cells, which might be exploited for experimental chemo-immunotherapy of cancer.

In recent years, attempts to xenogenize tumour cells have been pursued in a number of laboratories. It has been achieved by several procedures, including exposure to non-pathogenic viruses (Lindenman *et al.*, 1963; Kobayashi *et al.*, 1970), antitumour drugs (Bonmassar *et al.*, 1970; Nicolin *et al.*, 1972), through chemical haptenization (Martin *et al.*, 1971) or treatment with mutagens (Boon & Kellerman, 1977).

The antitumour agent, (5-(3,3′ dimethyl-l-triazeno)-imidazole-4-carboxamide (DTIC), employed in our experiments, has been found to be particularly effective in increasing the immunogenicity of certain experimental lymphomas (Bonmassar *et al.*, 1972; Nicolin *et al.*, 1976*a*).

The new antigenic specificities, not detectable on parental cells are retained as a genetic character after cessation of DTIC treatment (Nicolin *et al.*, 1974*a*) and induce an immune response in syngeneic hosts as evaluated by classical rejection experiments (Nicolin *et al.*, 1976*b*; Riccardi *et al.*, 1978) and by transfer of immune lymphocytes (Nicolin *et al.*, 1974*b*; Nicolin *et al.*, 1980; Romani *et al.*, 1983).

Furthermore, it has been shown that DTIC-altered tumour cells can elicit host resistance to a subsequent inoculum of untreated tumour cells (Campanile *et al.*, 1975; Nicolin *et al.*, 1981). In this respect DTIC treatment of tumour cells is likely to have some significance for immunotherapeutic approaches against neoplastic cells.

However, many fundamental questions related to the mechanism of immunological alterations and to the molecular characteristics and the functions, if any, of the DTIC-induced antigens remain unanswered.

The aim of this study was to further investigate antigenic changes detected in the tumour cell surfaces after DTIC treatment. We wished to know whether DTIC induces new antigen(s) (Bonmassar *et al.*, 1979) rather than amplifying a pre-existent tumour-associated antigen (TAA) (Mihich, 1969; Fuji *et al.*, 1979) or induces a product that behaves like a restriction element for the recognition of an antigen already expressed on untreated tumours (Hui *et al.*, 1984). Another purpose of this study was to determine the immunological relationship among genetically homogeneous L1210 cells, distinctly xenogenized through different cycles of DTIC treatments, using syngeneic cytolytic T-lymphocytes (CTL) and the derived clones.

### Materials and methods

### *Animals*

Hybrid (BALB/c $H-2^d \times DBA/2\ H-2^d)F_1$ (hereafter called $CD2F_1$) mice of both sexes, 6–8 weeks old were obtained from Charles River Breeding Laboratories (Calco, Italy).

### *Tumours*

L1210 Cr ($H-2^d$) (Law *et al.*, 1949; Dunham *et al.*, 1953) obtained from the National Cancer Institute (Bethesda, USA) and maintained in the laboratory by weekly i.p. injection into $CD2F_1$ mice was cloned *in vitro* by limiting dilution. Twenty cells from one clone (20 cells/4 ml) were injected (0.2 ml/mouse) into 20 syngeneic mice. Four of 8 subcloned tumour lines (referred to as A, D, R, S) were transformed *in vivo* with DTIC, as previously described (Bonmassar *et al.*, 1970). Briefly, $10^6$ tumour cells were injected i.p. into $CD2F_1$ mice treated for 7 consecutive days with DTIC ($100\,mg\,kg^{-1}\,day^{-1}$, i.p.) starting one day after tumour challenge. When the ascitic tumours developed in the DTIC-treated mice, they were collected and $10^6$ cells were transplanted into untreated mice. The same procedure was followed for 4–5 transplant generations.

Immunogenic subclones A/DTIC, D/DTIC, R/DTIC, S/

Correspondence: O. Marelli.
Received 8 January 1988; and in revised form, 12 March 1988.

DTIC were maintained in compatible, total body X-iirradiated (3.5 Gy; Securix Compact CGD), mice.

—YAC-1(H−2[a]) tissue culture cell line originating from YAC (Klein & Klein, 1964) was maintained in RPMI 1640 medium (Flow Lab.) supplemented with 10% heat-inactivated foetal calf serum (FCS) (Flow Lab.), $100\,U\,ml^{-1}$ penicillin, $100\,\mu g\,ml^{-1}$ streptomycin.

—P388 (H−2[d]) tumour cells (Dawe & Potter, 1957) were maintained by weekly i.p. injections into syngeneic DBA/2 mice.

*Preparation of T-cell growth factor (TCGF) from rat spleen cells*

Rat spleen cells ($5–10 \times 10^6$ cells $ml^{-1}$) were cultured for 24 h in RPMI 1640 medium supplemented with 10% heat inactivated FCS, $5 \times 10^{-5}$ M 2-mercaptoethanol, $100\,\mu\,ml^{-1}$ penicillin, $100\,\mu g\,ml^{-1}$ streptomycin and $2–5\,\mu g\,ml^{-1}$ Concanavalin A (Flow Lab.).

The supernatant that contained TCGF was collected by centrifugation, filtered and stored at −20°C.

*Cytotoxic T lymphocytes (CTL) and $^{51}Cr$ release assay*

Spleen cells from CD2F$_1$ mice injected 3–5 weeks before with $10^7$ DTIC treated subclones were stimulated secondarily *in vitro*.

$30 \times 10^6$ splenocytes (responders) were cultured in 25 cm$^2$ flasks (Sterilin) with $3 \times 10^6$ mitomycin C (Kiowa, Tokyo)-treated tumour cells (stimulators) at a final volume of 20 ml RPMI 1640 medium, supplemented as previously described. After 5 days incubation, the effector CTL were harvested and their lytic activity against different target cells was evaluated in a $^{51}Cr$ release microassay (Ferrini *et al.*, 1987).

Briefly, $^{51}Cr$-labelled target cells, $10^4$ in $100\,\mu l$, were seeded in 96 well plates (Sterilin) with $100\,\mu l$ effector cells at different concentrations and incubated for 4 h at 37°C in a moist atmosphere of 95% air and 5% $CO_2$. The plates were then centrifuged and $100\,\mu l$ supernatant counted in a gamma counter. Percent specific lysis was calculated as:

$$\%\ \text{lysis} = \frac{\text{cpm exp. release} - \text{cpm spontaneous release}}{\text{cpm max. release} - \text{cpm spontaneous release}} \times 100$$

Maximum release was obtained by incubating $10^4$ $^{51}Cr$ labelled target cells in $200\,\mu l$ Triton-X100 (1% in distilled water) for 4 h. Spontaneous release was obtained by incubating $10^4$ target cells with different concentrations of non-secondarily activated syngeneic splenocytes. The spontaneous $^{51}Cr$ release for the target cells used was in the range, 5–15%.

*CTL cloning*

Ten CTL, obtained as previously described, were placed in flat-bottom 96 well plates (Linbro Chemical Co.) under limiting dilution conditions (1, 10 and 100 cells per well) with $5 \times 10^4$ previously irradiated (60 Gy; $^{60}Co$) stimulator cells, $10^4$ irradiated (40 Gy; $^{60}Co$) syngeneic cells per well as feeders, and 10% TCGF.

The three plates seeded with 1 cell/well did not develop a colony, the 3 plates seeded with 10 cells/well developed a colony in 33% of the wells and when 100 cells were seeded colonies grew in almost all the wells. CTL clones used in this work derive from the plates seeded with 10 cells/well.

*Virus detection*

Cell culture supernatants were filtered ($0.2\,\mu m$ FlowPore D) and assayed for ecotropic murine retroviruses in mouse SC-1 cells by the XC plaque assay (Varnier & Levy, 1979; Rowe *et al.*, 1970).

No infectious virus ($\leq 4$ Plaque Forming Unit $ml^{-1}$) was detected in the samples.

The electron microscopic localization of MMTV antigens was performed by the pre-embedding protein A-gold technique.

### Results

Cloned cells from the mouse L1210 leukaemia, obtained by 'limiting dilution' *in vitro*, were inoculated (20 cells in 4 ml), i.p. into 20 syngeneic CD2F$_1$ mice (0.2 ml/mouse). Four of the 8 tumour subclones (A, D, R, S) that grew in the mice, were xenogenized *in vivo* by DTIC treatment, as previously described (Bonmassar *et al.*, 1970). Following 4–5 transplant generations the A/DTIC, D/DTIC, R/DTIC, S/DTIC sublines were fully xenogenized, since $10^7$ cells of each subclone were completely rejected by CD2F$_1$ mice. The experimental results in Table I show that xenogenized DTIC subclones retained the growth characteristics of the untreated L1210 parental subclones, since their tumorigenicity in immunosuppressed mice was fully retained.

The antigenic properties of the L1210/DTIC subclones and their reciprocal immunological relationships were studied with CTL. Spleen cells from CD2F$_1$ mice that had rejected $10^7$ xenogenized cells were secondarily restimulated *in vitro* with the relevant DTIC subclones. The results for the immunological cross-reactivities among L1210 DTIC subclones are listed in Table II.

In the 4 h $^{51}Cr$-release assay, anti-D/DTIC CTL had high lytic activity against D/DTIC target cells as well as against the other DTIC subclone lines. The parental, DTIC-untreated D subclone line was also lysed by anti-D/DTIC CTL, although the percentage of $^{51}Cr$ release was lower than that released by D/DTIC. The other untreated cell lines (A, R, S) were recognized and lysed to a lesser degree than D. There was detectable activity against YAC and P388 lymphomas. Similar patterns of cross-reactivity were displayed by anti-A/DTIC, anti-R/DTIC and anti-S/DTIC CTL (data not shown). Indeed the CTL to each xenogenized subclone were fully cross-reactive with the other DTIC treated subclones.

To further understand the antigenic properties of DTIC subclones, D/DTIC CTL were cloned in microtiter plates by limiting dilution and cultured in the presence of irradiated D/DTIC cells, syngeneic feeder spleen cells and rat IL-2. Three weeks later, it was possible to detect growing colonies in 94/288 microwells. Since the percentage of negative wells was ~67%, a high probability of cloning was ensured. The cytotoxic activity (lytic activity) of individual clones was assayed against the xenogenized cells, the untreated parental subclones and unrelated tumours. Twenty-one of the 94 CTL clones obtained had no lytic activity and 73 had cytotoxic activity. Among the 73 effective CTL clones, 36 had high cytotoxic activity against the 4 xenogenized subclone cells only (Table III), in contrast to the 12 CTL clones that had lytic activity against the L1210 subclone cells also (Table IV), whether xenogenized or not. None of the CTL clone groups had any effect on the unrelated tumour cells. Two CTL clones were weakly but highly specifically lytic to the

**Table I** Growth patterns of L1210 subclones in normal and immunosuppressed mice

| | *Intact mice* | | *Irradiated mice* | |
|---|---|---|---|---|
| *Tumour cells*[a] | *MST*[b] | *D/T*[c] | *MST* | *D/T* |
| D | 6.5 | 10/10 | 6 | 10/10 |
| D/DTIC | – | 0/10 | 6.5 | 10/10 |
| A | 7 | 10/10 | 6.5 | 10/10 |
| A/DTIC | – | 0/10 | 7 | 10/10 |
| R | 6.5 | 10/10 | 6 | 10/10 |
| R/DTIC | – | 0/10 | 7 | 10/10 |
| S | 7 | 10/10 | 7 | 10/10 |
| S/DTIC | – | 0/10 | 7.5 | 10/10 |

[a]$10^7$ viable cells/mouse, i.p.; [b]MST = mean survival time; [c]D/T = dead mice/treated mice; [d]3.5 Gy/mouse (day-1).

**Table II** Anti-D/DTIC CTL activity: percentage $^{51}Cr$ release (±s.d.)

| *Target cells* | *20:1*[a] | *10:1* | *5:1* | *2.5:1* | *1:1* |
|---|---|---|---|---|---|
| D/DTIC | 94.4±7.8 | 81.4±7.2 | 71.4±5.2 | 56.9±4.2 | 35.9±2.1 |
| D | 51.9±4.3 | 31.9±1.8 | 17.1±9.9 | 11.1±0.3 | 2.5±0.1 |
| A/DTIC | 43.9±3.1 | 34.6±1.9 | 28.5±1.1 | 23.7±1.2 | 17.2±0.7 |
| A | 15.0±1.2 | 9.6±0.5 | 7.7±0.4 | 2.7±0.1 | 0.2±0 |
| R/DTIC | 79.3±6.1 | 66.2±4 | 58.9±4.1 | 52.4±3.9 | 25.9±1.5 |
| R | 39.9±2.9 | 26.2±1.1 | 21.8±1.2 | 12.9±0.9 | 5.5±0.4 |
| S/DTIC | 84.3±7 | 73.4±5.1 | 64.4±4.2 | 48.9±3.1 | 25.9±1.9 |
| S | 24.9±1.9 | 19.0±0.1 | 17.0±1.2 | 5.6±0.3 | 2.1±0.1 |
| YAC | 14.2±0.9 | 9.9±0.5 | 2.6±0.1 | 1.6±0.2 | 0.4±0 |
| P388 | 19.4±1.2 | 9.6±0.8 | 9.2±0.4 | 6 ±0.4 | 3.4±0.2 |

[a]Effector: target cell ratio.

**Table III** Anti-D/DTIC CTL clones: Specific lysis of DTIC subclones

| *Target cells* | *Clone no.* | | | | |
|---|---|---|---|---|---|
| | *11* | *14* | *33* | *69* | *85* |
| D | 3.2±0.2[a] | 0.6±0 | 0.8±0.1 | 8.1±0.7 | 7.0±0.3 |
| D/DTIC | 83.9±6.2 | 78.9±6.9 | 62.5±3.9 | 87.3±7.1 | 82.9±7.2 |
| A | 2.1±0.1 | 0.5±0 | 3.2±0.5 | 8.2±0.9 | 2.3±0.4 |
| A/DTIC | 81.4±7 | 84.4±7.1 | 71.4±4 | 79.4±6.2 | 78.7±5.9 |
| R | 7.3±0.7 | 3.5±0.4 | 6.9±0.6 | 0.3±0 | 2.1±0.1 |
| R/DTIC | 77.9±6.4 | 79.5±5.2 | 84.1±6.2 | 69.5±5.9 | 71.9±6 |
| S | 5.3±0.4 | 5.3±0.5 | 0.2±0 | 3.2±0.3 | 6.4±0.6 |
| S/DTIC | 80.5±7.1 | 80.2±7 | 72.7±4.9 | 68.9±5.6 | 79.8±6.1 |
| YAC | 4.2±0.3 | 2.1±0.3 | 11.2±0.7 | 0.6±0 | 6.5±0.5 |
| P388 | 7.7±0.6 | 3.4±0.2 | 0.2±0 | 9.3±0.8 | 8.2±0.6 |
| EL4 | 1.1±0 | 1.2±0 | 10.3±0.7 | 7.2±0.5 | 3.4±0.1 |

Similar activities were displayed by 31 CTL clones not reported here; [a]% $^{51}Cr$ release±s.d.; Effector:target cell 5:1.

**Table IV** Anti-D/DTIC CTL clones: Lysis of L1210 subclones

| *Target cells* | *Clone no.* | | | | |
|---|---|---|---|---|---|
| | *6* | *16* | *48* | *52* | *78* |
| D | 68.5±3.1[a] | 62.6±4.5 | 73.5±6.2 | 57.2±3.9 | 81.9±7 |
| D/DTIC | 71.8±4 | 66.7±3.2 | 68.4±4.7 | 80.4±7 | 58.2±3.2 |
| A | 73.6±3.9 | 78.2±5.1 | 58.0±3.2 | 56.3±4 | 76.6±5.2 |
| A/DTIC | 64.7±2.2 | 68.9±4 | 76.6±5.2 | 59.2±3.9 | 69.4±5 |
| R | 59.6±2.8 | 78.7±5.4 | 74.4±6.4 | 66.5±4.1 | 74.9±5.9 |
| R/DTIC | 77.2±4.6 | 73.9±4.2 | 56.3±4.2 | 72.9±4.3 | 62.1±4.2 |
| S | 61.5±3.4 | 68.5±3.9 | 77.2±5.9 | 62.9±3.9 | 81.1±6.9 |
| S/DTIC | 65.6±4.2 | 70.6±5.2 | 74.9±6 | 63.7±3.8 | 63.2±4.3 |
| YAC | 4.3±0.1 | 8.3±0.6 | 2.3±0.1 | 5.4±0.6 | 0.4±0 |
| P388 | 3.2±0.2 | 0.2±0 | 8.4±0.4 | 7.2±0.7 | 6.3±0.6 |
| EL4 | 1.3±0 | 8.9±0.5 | 2.9±0.2 | 4.3±0.2 | 3.2±0.1 |

Similar activities were displayed by 7 CTL clones not reported here; [a]% $^{51}Cr$ release±s.d.; Effector:target cell 5:1

**Table V** Anti-D/DTIC CTL clones: Specific lysis (%±s.d.) of D subclones

| *Target cells* | *Clone no.* | |
|---|---|---|
| | *32* | *57* |
| D | 28.3±1.5[a] | 34.3±1.6 |
| D/DTIC | 39.4±1.9 | 31.7±2 |
| A | 0.4±0 | 2.0±0.2 |
| A/DTIC | 4.0±0.1 | 0.3±0 |
| R | 3.2±0.1 | 2.3±0.1 |
| R/DTIC | 5.1±0.4 | 1.1±0.1 |
| S | 2.2±0.3 | 0.6±0 |
| S/DTIC | 5.3±0.6 | 2.5±0.3 |
| YAC | 5.2±0.7 | 0.8±0 |
| P388 | 7.1±0.9 | 2.6±0.4 |
| EL4 | 4.0±0.1 | 1.7±0.5 |

[a]% $^{51}Cr$ release; Effector:Target cell 5:1.

**Table VI** Recognition pattern of anti-D/DTIC CTL clones

| *Target cells* | *CTL clone groups of activity* | | |
|---|---|---|---|
| | *I* | *II* | *III* |
| D/DTIC | +++ | +++ | + |
| D | − | +++ | + |
| A/DTIC | +++ | +++ | − |
| A | − | +++ | − |
| R/DTIC | +++ | +++ | − |
| R | − | +++ | − |
| S/DTIC | +++ | +++ | − |
| S | − | +++ | − |
| YAC | − | − | − |
| P388 | − | − | − |
| EL4 | − | − | − |

Note: +++ 60–80% specific $^{51}Cr$ release; + 30% specific $^{51}Cr$ release.

D/DTIC subclone and its corresponding D parental subclone (Table V). The specific activity of CTL clones could therefore be differentiated into three groups, (Table VI). The last 23 CTL clones had no specific activities, since they showed different patterns of reactivity. All of them were able to lyse the L1210 derived lines, YAC cells and different non-related tumour cell lines (P388, L5178Y, EL4), but the degree of lysis of each target cell was different, clone by clone (data not shown). The pattern of activity of these clones is the pattern of reactivity described for activated NK (Henney et al., 1981; Suzuki et al., 1983) and/or LAK cells (Rosenstein et al., 1984; Merluzzi, 1984).

### Discussion

This laboratory has been involved in attempts to elucidate the immunological alterations in L1210 leukaemia after treatment with the anticancer agent DTIC. Briefly, DTIC-treated L1210 cells are rejected by immunocompetent syngeneic animals, even after very heavy cell challenges in spite of the fact that these cells show the same kinetic properties as those of parental L1210 cells both *in vitro* and in immunodeficient hosts (Silvestrini et al., 1977). The modification at the molecular level is still unknown. Studies of DTIC-induced antigenicity and of similar immunological alterations produced in different tumour cells by other anticancer compounds (Mihich, 1969; Nicolin et al., 1972; Frost, 1984), or mutagenic agents (Boon, 1983; Altevog et al., 1985), have been hampered by difficulties in raising effective polyclonal or monoclonal antibodies. Further characterization of DTIC antigens might be useful in shortening the time schedule required to xenogenize the tumour cells, in order to achieve the tumour rejection in primary hosts. Furthermore the DTIC-induced antigens might be exploited for active vaccination or passive immunotherapy.

Previous studies with cell-mediated cytotoxic assays from this laboratory (Marelli et al., 1986) have excluded the likelihood that DTIC positively selects naturally occurring non-tumorigenic cells and indicate that there is a limited number of antigens in DTIC-treated L1210 cells. The number of antigens expressed by DTIC-treated L1210 cells is still not exactly defined because of the genetic heterogeneity of the tumour populations.

In this study, we found that four L1210 subclones, submitted separately to DTIC treatments, were not tumorigenic in syngeneic hosts although they were as tumorigenic as untreated subclones in immunodepressed mice. The genetic homogeneity within the four subclones, rendered it impossible for DTIC to select preexisting non-tumorigenic variant cells from the L1210 subclones. Therefore, we assume that the antigenic properties of treated L1210 subclones were directly altered by DTIC treatment.

Serological studies carried out in collaboration with the Tissue Typing Research Laboratories of the London Hospital Medical College (Marelli & Kimura, unpublished observations) provide no evidence for qualitative and/or quantitative differences of MHC class I antigen expression in the parental and DTIC treated cells. All the subclones (DTIC treated and untreated), as well as the L1210 line from which they were derived, were negative for MHC class II antigen expression.

It was therefore of interest to establish the number of antigens expressed on each DTIC subclone as well as to study the immunological relationships among the subclones. Since all four CTL populations lysed all four DTIC subclones, the immunological cross-reactivity of the DTIC subclones was proven. However, parental subclones and NK sensitive YAC cells were also lysed although less efficiently. Therefore, we have not established the number of DTIC antigenic specificities on the cells, not whether the DTIC subclones expressed new antigens or overexpressed the L1210 TAA putatively responsible for the partial CTL cross reactivity. Moreover the possibility that the increased antigenicity was due to the induction of a product that behaves like a restriction element for the recognition of an antigen already expressed on untreated tumour cells, could not be ruled out. These questions were approached by testing the CTL clones obtained from the anti-D/DTIC CTL population against the battery of relevant target cells. As shown in Table VI, three types of specific antigens could be identified. A number of CTL clones were able to effectively lyse only the DTIC subclones.

These results concur with the hypothesis that a new antigen (or set of antigens) is induced by DTIC treatment. To date, every property related to the drug treatment and expressed on D/DTIC cells is shared by all the other DTIC-modified lines. However, the design of our studies does not exclude in principle that within A/DTIC, R/DTIC or S/DTIC cell populations there might be other products not shared with the D/DTIC cell population. Likewise, within D/DTIC there might be cells carrying determinants unrecognized by the CTL clones studied here and unique for D/DTIC lines. Investigations relating to virus infection have excluded differences among parental and DTIC subclones. All L1210 tumour lines and P388 used here were positive for mammary tumour virus (Squartini & Marchetti, unpublished data), as determined in an immunocytochemical study with rabbit anti-MTV serum and protein A-gold complexes, while they were negative for type C viruses (Varnier, unpublished data) in plaque assays. On the basis of these observations we can postulate the existence of a reproducible and specific DTIC-induced antigen responsible for genetic and phenotypic xenogenization of L1210 leukaemia.

The biochemical structure of the DTIC induced antigen is unknown. This study shows that the TAA of L1210 is not functionally modified, since a set of cytotoxic cloned CTL did not discriminate between parental and DTIC-treated cells. This antigen(s) cannot be responsible for host rejection, since challenge with as few as 10 cells of parental subclones was lethal. Although it is quite likely that this common determinant is comparable with a TAA expressed on L1210, since P388 and YAC cells are not recognized, we cannot rule out the possibility that these cross-reactivities might be due to the presence of viral products (Racevskis & Sarkar, 1982; Zac-Nejmark et al., 1978).

Two CTL clones showed weak but highly specific reactivity against the immunizing target (D/DTIC) cells and the respective untreated lines (D). Although the parental cell lines D, A, R, S were derived from one clone of L1210, a mutational event occurred before the DTIC treatment could be responsible for the induction of a determinant on the untreated D cell line that is retained on D/DTIC cells. The antigens recognized by the 23 clones that behave in a nonspecific way could belong to the category of molecules that are targets for LAK or activated NK.

We did exclude that they are classical NK targets, since L1210 cells (Fuji & Iribe, 1986) were not NK sensitive. Moreover the parental and DTIC treated subclones involved in this study were not lysed in classical NK experiments (data not shown).

Little is known so far about the mechanism by which alkylating compounds, such as DTIC (Audette et al., 1973), have such a strong effect on the immunogenicity of tumour cells. Since DTIC treatment has to be protracted to obtain tumour lines rejected by a syngeneic host, it is quite likely that more than one mutational event occurs, but probably only a few of these lead to increased antigenicity. Furthermore, our results suggest that DTIC induces mutations, leading to the recurrent expression of new antigens at preferential sites.

Studies are in progress to elucidate the biochemistry and genetics of DTIC induced-antigens.

Whatever the chemical structure of the DTIC-induced antigens, the data presented in this paper may prove useful for a chemo-immunological approach to tumour therapy.

Research in part supported by Progetto Finalizzato Oncologia, CNR, Rome, Italy and by a grant from AIRC. (to O.M.). N. Prandoni is a recipient of a grant from AIRC.

We thank Prof. F. Squartini and A, Marchetti for the MTV assays and Prof. O. Varnier for the type C virus assays.

### References

ALTEVOG, T., VON MOEGEN, P., LEIDIG, S. & SHIRRMACHER, V. (1985). Effect of mutagens on the immunogenicity of murine tumor cells: Immunological and biochemical evidence for altered cell surface antigens. *Cancer Res.*, **45**, 4270.

AUDETTE, R.C.S., CONNORS, T.A., MANDEL, H.G., MERAI, K. & ROSS, W.C.J. (1973). Studies on the mechanism of the tumour inhibitory triazenes. *Biochem. Pharmacol.*, **22**, 1855.

BONMASSAR, E., BONMASSAR, A., VLADAMUDI, S. & GOLDIN, A. (1970). Immunological alteration of leukemic cells *in vivo* after treatment with an antitumor drug. *Proc. Natl Acad. Sci. USA.*, **66**, 1089.

BONMASSAR, E., BONMASSAR, A., VLADAMUDI, S. & GOLDIN, A. (1972). Antigenic changes of L1210 leukemia in mice treated with 5-(3,3 dimethyl-l-triazeno) imidazole-4-carboxamide. *Cancer Res.*, **32**, 1446.

BONMASSAR, E., NICOLIN, A. & SPREAFICO, F. (1979). Drug induced modification of tumor cell antigenicity. In *Tumor associated antigens and their specific immune response*, Spreafico, F. & Arnon, R. (eds). Academic Press: New York.

BOON, T. & KELLERMAN, O. (1977). Rejection by singeneic mice of cell variants obtained by mutagenesis of a malignant teratocarcinoma cell line. *Proc. Natl Acad. Sci. USA.*, **74**, 272.

BOON, T. (1983). Antigenic tumor cell variants obtained with mutagens. *Adv. Cancer Res.*, **39**, 121.

CAMPANILE, F., HOUCHENS, D.P., GASTON, M., GOLDIN, A. & BONMASSAR, E. (1975). Increased immunogenicity of two lymphomas lines after drug treatment of athimic (nude) mice. *J. Natl Cancer Inst.*, **55**, 207.

DAWE, C.J. & POTTER, M. (1957). Morphologic and biologic progression of a lymphoid neoplasm of the mouse *in vivo* and *in vitro*. *Amer. J.. Path.*, **33**, 603.

DUNHAM, L.J. & STEWART, H.L. (1953). A survey of transplantable and transmissible animal tumors. *J. Natl Cancer Inst.*, **13**, 1299.

FERRINI, S., MIESCHER, S., ZOCCHI, M.R., VON FLIEDNER, V. & MONTTA, A. (1987). Phenotypic and functional characterization of ricombinant Interleukin 2 induced activated killer cells. Analysis at the population and clonal level. *J. Immunol.*, **138**, 1297.

FROST, P., LITEPLO, R.G., DONAGHUE, T.P. & KERBEL, R.S. (1984). Selection of strongly immunogenic tum- variants from tumors at high frequency using 5-azacytidine. *J. Exp. Med.*, **159**, 1491.

FUJI, H., MIHICH, E. & PRESSMAN, D. (1979). Differential tumor immunogenicity of DBA/2 mouse lymphoma L1210 and its sublines. II. Increased expression of tumor-associated antigens on subline cells recognized by serologic and transplantation methods. *J. Natl Cancer Inst.*, **62**, 1503.

FUJI, H. & IRIBE, H. (1986). Clonal variation in tumorigenicity of L1210 lymphoma cells: Non tumorigenic variants with enhanced expression of tumor associated antigen and Ia antigens. *Cancer Res.*, **46**, 5541.

HENNEY, C.S., KURIBAYASHI, K., KERN, D. & GILLIS, S. (1981). Interleukin-2 augments natural killer cell activity. *Nature*, **291**, 335.

HUI, K., GROSVELD, F. & FESTENSTEIN, H. (1984). Rejection of transplantable AKR leukemia cells following MHC DNA-mediated cell transformation. *Nature*, **311**, 750.

KLEIN, E. & KLEIN, G. (1964). Antigenic proprieties of lymphoma induced by the Moloney agent. *J. Natl Cancer Inst.*, **32**, 547.

KOBAYASHI, M., SENDO, F., KAJI, H. & 5 others (1970). Inhibition of transplanted rat tumors by immunization with identical tumor cells injected with Friend virus. *J. Natl Cancer Inst.*, **44**, 11.

LINDENMAN, J. & KLEIN, P. (1963). Viral oncolysis: Increase in immunogenicity of host cell antigen associated with influenza virus. *J. Exp. Med.*, **126**, 93.

LAW, L.W., DUNN, T.B., BOYLE, P.J. & MILLER, H.J. (1949). Observations on the effect of a golic-acid antagonist on transplantable lymphoid leukemias in mice. *J. Natl Cancer Inst.*, **10**, 179.

MARELLI, O., CANTI, G., FRANCO, P., PRANDONI, N., RICCI, L. & NICOLIN, A. (1986). L1210/DTIC antigenic subline: Studies at the clone level. *Eur. J. Cancer Clin. Oncol.*, **22**, 1401.

MARTIN, W.J., WÜNDERLICH, J.R., FLETCHER, F. & IMMAN, J.K. (1971). Enhanced immunogenicity of chemically coated syngeneic tumor cells. *Proc. Natl Acad. Sci. USA.*, **68**, 469.

MERLUZZI, V.J., SAVAGE, D.M., MERTELSMANN, R. & WECTE, K. (1984). Generation of non specific murine cytotoxic T-cells *in vitro* by purified human interleukin-2. *Cell Immunol.*, **84**, 74.

MIHICH, E. (1969). Modification of tumor regression by immunological means. *Cancer Res.*, **29**, 2345.

NICOLIN, A., VLADAMUDI, S. & GOLDIN, A. (1972). Antigenicity of L1210 sublines induced by drugs. *Cancer Res.*, **37**, 653.

NICOLIN, A., BINI, A., CORONETTI, E. & GOLDIN, A. (1974*a*). Cellular immune response to a drug treated L5178Y lymphoma subline. *Nature*, **251**, 654.

NICOLIN, A., CANTI, G. & GOLDIN, A. (1974*b*). Adoptive immunotherapy in Balb/c x DBA/2Cr $F_1$ mice bearing an immunogenic subline of L1210 leukemia. *Cancer Res.*, **34**, 3044.

NICOLIN, A., SPREAFICO, F., BONMASSAR, E. & GOLDIN, A. (1976*a*). Antigenic changes of L5178Y lymphoma after treatment with 5-(3,3 dimethyl-l-triazeno) imidazole-4-carboxamide *in vitro*. *J. Natl Cancer Inst.*, **58**, 89.

NICOLIN, A., FRANCO, P., TESTORELLI, C. & GOLDIN, A. (1976*b*). Immunosensitivity and histocompatibility antigens in drug-altered leukemic cells. *Cancer Res.*, **36**, 222.

NICOLIN, A., VERONESE, F., MARELLI, O. & GOLDIN, A. (1980). Immunological resistance to L1210 leukemia induced by viable L1210/DTIC cells. *Cancer Immunol. Immunother.*, **9**, 43.

NICOLIN, A., CANTI, G., MARELLI, O., VERONESE, F. & GOLDIN, A. (1981). Chemotherapy of L1210 leukemic mice with antigenic tumor sublines. *Cancer Res.*, **41**, 1358.

RACEVSKIS, J. & SARKAR, N.H. (1982). ML antigen of DBA/2 mouse leukemias: Expression of an endogenous murine mammary tumor virus. *J. Virology*, **42**, 804.

RICCARDI, C., FIORETTI, M.C., GIAMPIETRI, A., PUCCETTI, P. & GOLDIN, A. (1978). Growth and rejection patterns of murine lymphoma cells antigenically altered following drug treatment *in vivo*. *Transplantation*, **25**, 63.

ROMANI, L., BIANCHI, R., PUCCETTI, P. & FIORETTI, M.C (1983). Systemic adoptive immunotherapy of highly immunogenic lymphoma growing in the brain. *Int. J. Cancer*, **31**, 477.

ROSENSTEIN, M., YRON, I., KAUFMANN, Y. & ROSENBERG, S.A. (1984). Lymphokine activated killer cells: Lysis of fresh syngeneic natural killer-resistant murine tumor cells by lymphocytes cultured in interleukin-2. *Cancer Res.*, **44**, 1946.

ROWE, W.B., PUGH, W.E. & HARTLEY, J.W. (1970). Plaque assay techniques for murine leukemia viruses. *Virology*, **42**, 1136.

SILVESTRINI, R., TESTORELLI, C., GOLDIN, A. & NICOLIN, A. (1977). Cell kinetics and immunogenicity of lymphoma cells treated with 5-(3,3′ dimethyl-l-triazeno) imidazole-4-carboxamide (DIC) *in vivo*. *Int. J. Cancer*, **19**, 644.

SUZUKI, R., HANDA, K., ITOH, K. & KUMAGAI, K. (1983). Natural killer (NK) cells as responders to interleukin-2 (IL-2). Proliferative response and establishment of cloned cells. *J. Immunol.*, **130**, 981.

VARNIER, O.E. & LEVY, J.A. (1979). Differential effect of Dexamethazone on replication of ecotropic and xenotropic mouse type C virus. *Virology*, **96**, 604.

ZAC-NEJMARK, T., STEUDEN, J. & RADZIKOWSKI, C. (1978). Mammary leukemia (ML) antigen isolated from L1210 leukemia cells. *Int. J. Cancer*, **21**, 490.